# A novel homozygous *HES7* splicing variant causing spondylocostal dysostosis 4: a case report

**DOI:** 10.3389/fped.2023.1201999

**Published:** 2023-08-25

**Authors:** Shaoguang Lv, Yuanyuan Wu, Fang Liu, Baoquan Jiao

**Affiliations:** ^1^ Department of Pediatrics, Bethune International Peace Hospital, Shijiazhuang, China; ^2^ Department of Reproduction and Genetics, Bethune International Peace Hospital, Shijiazhuang, China

**Keywords:** spondylocostal dysostosis 4 (SCDO4), *HES7* gene, alternative splicing mutation, minigene analysis, case report

## Abstract

**Background:**

Spondylocostal dysostosis 4 (SCDO4) is characterized by short stature (mainly short trunk), dyspnea, brain meningocele, and spina bifida occulta, which is caused by homozygous or compound heterozygous *HES7* (HES family bHLH transcription factor 7) variants. The incidence of SCDO4 remains unknown due to the extremely low number of cases. This study reveals a novel homozygous *HES7* splicing variant causing SCDO4 and reviews all the previously reported *HES7* variants and corresponding symptoms, providing a comprehensive overview of the phenotypes and genotypes of *HES7* variants.

**Case presentation:**

This case report focuses on a Chinese neonate who was first hospitalized for tachypnea, cleft palate, and short trunk. After a series of auxiliary examinations, the patient was also found to have deformities of vertebrae and rib, left hydronephrosis, and patent foramen ovale. He underwent surgery for congenital hydronephrosis at 5 months old and underwent cleft palate repair when he was 1 year old. After two and half years of follow-up, the boy developed normally. A novel homozygous *HES7 splicing* variant (c.226+1G>A, NM_001165967.2) was identified in the proband by whole-exome sequencing and verified by Sanger sequencing. The variant was inherited from both parents and minigene assays demonstrated that this variant resulted in the retention of intron3 in the *HES7* transcript. Including this case, a total of six *HES7* variants and 13 patients with SCDO4 have been reported.

**Conclusions:**

Our findings expand the genotype-phenotype knowledge of SCDO4 and provide new evidence for genetic counseling.

## 1. Introduction

Spondylocostal dysostosis (SCD) is a rare genetic disease that was first reported by McAlister in 1973 (1); however, the incidence remains unknown. It is characterized by widespread hemivertebrae, shortening of the trunk, and abnormal rib arrangement, which are caused by interruption of the spine, ribs, related tendons, and muscle precursor tissues during embryonic development. In total, six genes have been reported to be involved in the pathogenesis of SCD, namely, delta-like canonical Notch ligand 3 (*DLL3*), mesoderm posterior bHLH transcription factor 2 (*MESP2*), LFNG O-fucosylpeptide 3-beta-N-acetylglucosaminyltransferase (*LFNG*), HES family bHLH transcription factor 7 (*HES7*), T-box transcription factor 6 (*TBX6*), and ripply transcriptional repressor 2 (*RIPPLY2*). Therefore, hypoplasia of the spine and ribs is divided into six types, based on the various mutated genes (2, 3). Among them, homozygous or compound heterozygous variants of the *HES7* gene cause spondylocostal dysostosis 4 (SCDO4, #613686), which was first reported by Sparrow in 2008 (4).

The clinical manifestations of SCDO4 include intrauterine growth retardation, short stature (mainly short trunk), short chest, dyspnea, brain meningocele, spina bifida occulta, and so on. Imaging examination may reveal hemivertebrae, butterfly vertebrae, rib fusion, spinal canal abnormalities, spinal segment defects or non-segmentation, and heart and large blood vessel malformations. To date, only six SCDO4 variants have been reported (4–8). Five of them were found in the coding region, whereas one was located in the 3′-untranslated region (UTR) of the *HES7* gene. This article reports a novel homozygous *HES7* splicing variant causing SCDO4 in a Chinese neonate. The findings expand the genotype and phenotype spectrum of *HES7* variants.

## 2. Case report

### 2.1. Case presentation and follow-up

The proband was a Chinese full-term boy who was first hospitalized for tachypnea, cleft palate, and short trunk shortly after birth. The gestational age was 38 + 2 weeks and the birth weight was 2,850 g. The Apgar score was 9 points for 1 min, 5 min, and 10 min (all were breathing-1 scores). His mother was healthy during the pregnancy and had no history of pregnancy-induced hypertension, diabetes, or viral infection. His parents were unrelated and denied any family history of genetic diseases. The proband was the second child of these unrelated parents, who had a healthy older daughter. Ultrasound examination in the third trimester of pregnancy showed spinal column disorder, low position of the spinal cord, unclear display of the aortic arch, and bilateral renal pelvis separation.

Physical examination on admission: heart rate 140 beats/min, respiration 50 breaths/min, SpO_2_ 92% (oxygen concentration 30%), blood pressure 65/32 mmHg, body weight 2,850 g (10–25th percentile), head circumference 33 cm (10th percentile), and body length 42 cm (<3rd percentile). The proband's cry was loud and the response was good. A positive inspiratory trident sign was observed. There was no jaundice, cyanosis, or other skin abnormalities. The soft palate and part of the hard palate were cracked in the oral cavity. The thorax was short, but no pathological murmur was heard on auscultation. The abdomen was soft, and the liver and spleen were unpalpable under the ribs. The primitive reflexes were intact.

**Figure 1:**
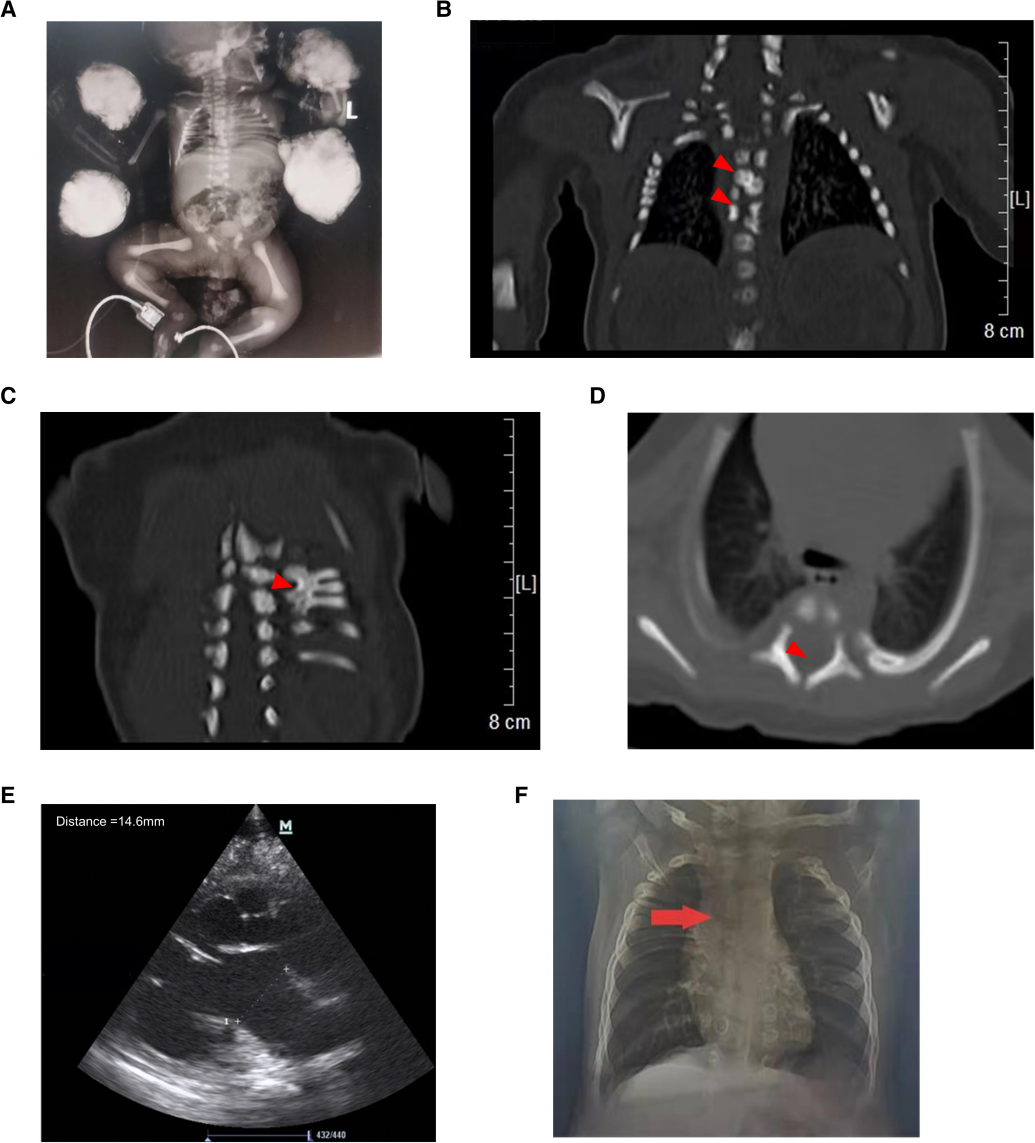
Abnormal imaging of the proband. (**A**) X-ray of the proband showing a short chest and distorted and fused ribs. Chest CT demonstrating hemivertebrae (**B**, upper arrow), butterfly vertebrae (**B**, lower arrow), rib fusion (**C**), and spinal canal enlargement (**D,E**) Kidney color Doppler ultrasound showing left hydronephrosis. (**F**) Chest x-ray showing mild scoliosis at the age of 11 months.

Auxiliary examinations: routine laboratory testing was normal. Chest x-ray confirmed a short rib cage, with a length of approximately 3.4 cm, showing distorted and fused ribs (Figure 1A). Chest CT demonstrated skeletal deformities, including half cones, butterfly vertebrae, rib fusion, and spinal canal enlargement (Figures 1B–D). Kidney color Doppler ultrasound revealed left hydronephrosis (approximately 13 mm), and right renal collection system separation (approximately 4.9 mm) (Figure 1E). Furthermore, a patent foramen ovale was found on cardiac color Doppler ultrasound. No abnormalities were reported by cranial ultrasound and magnetic resonance.

After 44 days of treatment with special pacifier feeding, anti-infection agents, and oxygen support, the child was discharged from the hospital and sucked well without choking. At the 3-month follow-up, the child sucked well without choking but still showed poor growth, with a body weight of 4,530 g (<3rd percentile), head circumference of 37 cm (<3rd percentile), and body length of 59 cm (10th percentile). A mild inspiratory three-concave sign was observed with SpO_2_ 95% (on room air). He underwent surgery for congenital hydronephrosis at 5 months old and underwent cleft palate repair when he was 1 year old. At 1 year old, his body weight was 7,280 g (3rd percentile), with a head circumference of 43.5 cm (3rd–10th percentile), and a body length of 70 cm (3rd percentile). The boy could stand while holding things and could pronounce “Dad, Mom”. He had a good diet without coughing after eating rice porridge and drinking milk. At this age, the inhalation three-concave sign was negative. Mild scoliosis was observed on the chest x-ray (Figure 1F). At the age of 18 months, the body weight was 9,000 g (3rd percentile), the head circumference was 45.5 cm (3rd percentile), and the body length was 77 cm (3rd percentile). The proband could walk alone and could speak words clearly. The scoliosis showed no progression. Due to the absence of other abnormal symptoms, no treatment was given. At the time of submitting this manuscript, the child was 2 years and 7 months old with a body weight of 12,400 g (25–50th percentile), head circumference of 48.5 cm (25–50th percentile), and body length of 89 cm (10th percentile). He could run and play normally, distinguish colors and partial shapes, and express his meaning in sentences, such as “Mom, we play hide-and-seek” etc. The scoliosis still did not progress, but the boy was required to attend regular follow-ups. A developmental assessment was performed using a neurodevelopmental scale for children aged 0–6 years. The results were gross motor 30, fine motor 33, adaptive ability 31.5, language 30, social behavior 30, intellectual age 30.9, developmental quotient 99.6, and moderate intelligence. No adverse or unanticipated events occurred. The parents were satisfied with the development of the proband. Long-term follow-up was needed for further observation.

### 2.2. Genotype and phenotype spectrum of *HES7* variants

Spondylocostal dysostosis may be caused by at least six distinct gene variants. Whole-exome sequencing (WES) and copy number variation (CNV) detection were conducted to discover the pathogenic genetic variations responsible for the symptoms in the proband. Written informed consent was obtained from the patient's parents. This project was approved by the Ethics Committee of Bethune International Peace Hospital (Approval Nos. 20180023 and 2022-KY-26). Trio-WES and trio-CNV detection were performed on an Illumina Novaseq6000 sequencing system in Beijing Berry Hekang Medical Laboratory. Sequencing data were compared with the human reference genome (hg19/GRCh37) using Burrows–Wheeler Aligner (BWA, CA, USA) software to align the original mapping result. Picard was used to mark and remove duplicate reads. Based on the results of the alignments, the obtained set of candidate variants was functionally annotated by Variant Effect Predictor (VEP) using a variety of bioinformatic databases. In brief, the detected variants fulfilled the following strategies: variants of single-nucleotide polymorphisms, splicing-sites variants, inframe indels, and frameshift insertions and deletions; variants with a minor allele frequency <0.5% in the genome Aggregation Database (gnomAD), 1,000 Genomes Project, Exome Aggregation Consortium (ExAC), or Exome Sequencing Project (ESP6500si); the retained missense variants were submitted to PolyPhen-2, SIFT or M-CAP for functional prediction and scored as “deleterious” by SIFT, “damaging” or “possibly damaging” by Polyphen-2, and “probably damaging” or “possibly pathogenic” by M-CAP. CNVs were evaluated by an in-house pipeline using read counts based on a smoothness model (Berry Genomics, Beijing, China) according to a previous study (9). The pathogenicity of each variant was assessed according to the American College of Medical Genetics and Genomics (ACMG) guidelines (10, 11).

**Figure 2:**
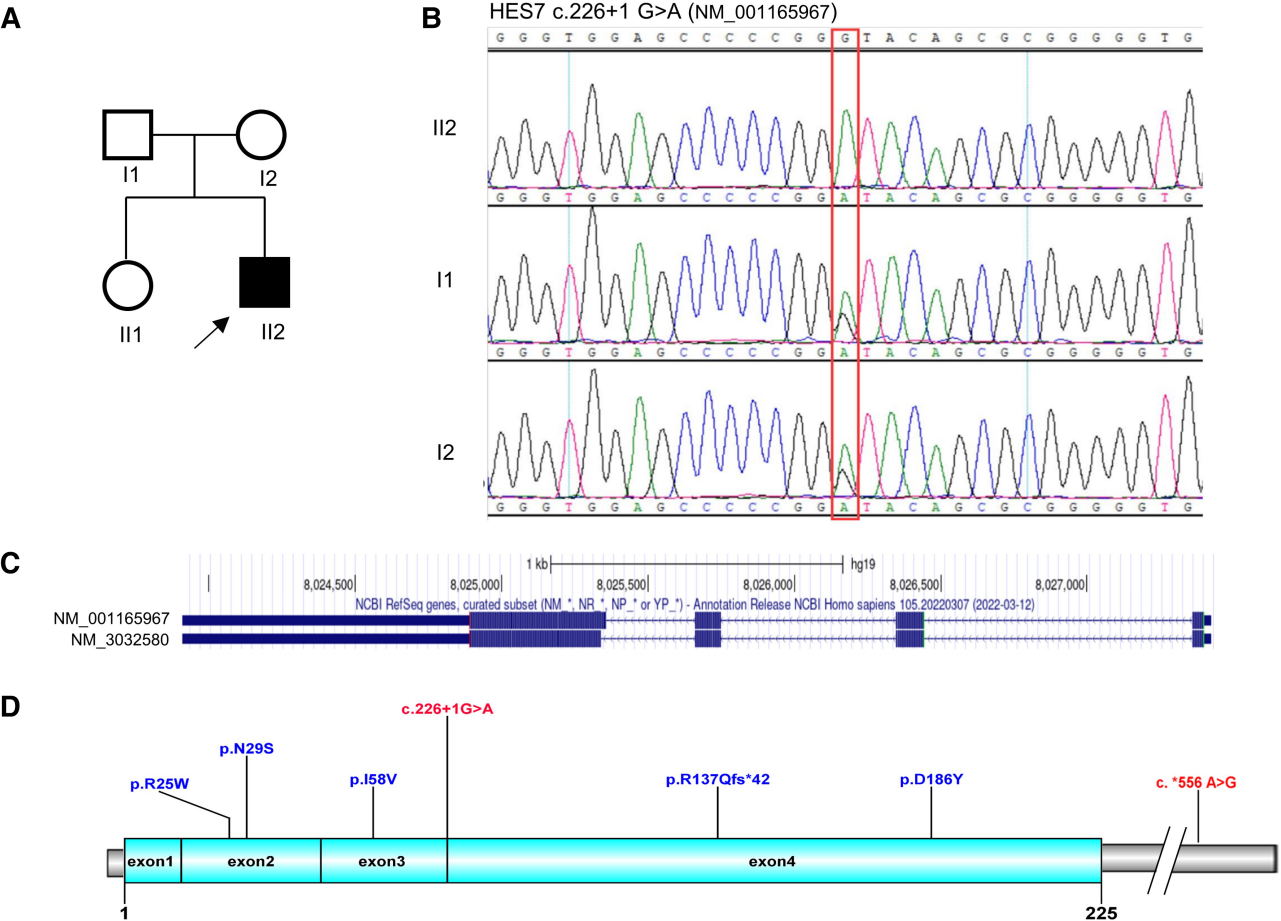
Genetic analysis of the *HES7* gene. (**A**) Pedigree diagram and (**B**) Sanger sequencing results of the family. (**C**) Transcripts of the *HES7* gene. (**D**) Overview of the reported *HES7* variants.

A homozygous variant *HES7* c.226+1G>A was identified by trio-WES, which was inherited from both parents (Figures 2A,B). This novel variant was located at the splicing region, which was predicted to influence the splicing of *HES7* mRNA (PVS1). Nevertheless, this variant was not found in reference databases such as 1,000 genomes, ExAC, gnomAD, or the local Berry database with only Chinese samples (PM2_supporting). As the parents of the proband denied consanguinity, kinship analysis was performed using King software with WES data (12). The kinship value between the couple was 0.0721 which indicated that they were third degree relatives (Supplementary Figure S1). The couple have no common relatives, but they come from the same town. Founder effect may be an explanation for this non-consanguineous couple carrying the same rare variant. No suspicious variants were found in the trio-CNV detection results. The proband was finally diagnosed as SCDO4 based on the WES results.

*HES7* is located at chromosome 17p13.1 and has two transcripts, namely, NM_001165967 and NM_3032580 (Figure 2C). The two transcripts share the same transcription initiation point, transcription endpoint, and exon number. The only difference between the two transcripts is that the fourth exon of NM_001165967 has five more amino acids than that of NM_3032580. By the end of April 2023, only six pathogenic variants of *HES7* have been reported in public databases (Figure 2D, Table 1), including four missense variants, one frameshift variant, and one variant in the 3′-UTR. These variants were distributed dispersedly in the whole *HES7* region. Including the patient in this article, a total of 13 cases have been reported with detailed clinical symptoms (Table 1). Overall, 84.6% (11/13) of patients showed short stature, 53.8% (7/13) had dextrocardia, 30.8% (4/13) presented vertebral artery hypoplasia, 83.3% (10/12) presented a restrictive ventilatory defect, and 84.6% (11/13) demonstrated chest deformities such as situs inversus, short thorax, rib deformity, and pectus excavatum. Furthermore, 23.1% (3/13) of patients presented abdomen situs inversus, 61.5% (8/13) exhibited spine abnormities such as hemivertebrae, butterfly vertebra, and abnormal odontoid process, and 50% (6/12) showed spina bifida occulta or myelomeningocele. 76.9% (10/13) of patients were alive when their case was reported (Table 1). The proband in this report presented short stature, short thorax, rib and spine deformities, which are the typical symptoms of SCDO4 (PP4).

**Table 1:** Summary of *HES7* gene variants and detailed symptoms.

Patients		c. 400_409dupAAACCGCCCC, p.R137Qfs *42							c.73C>T p.R25W	c.172A>G p.I58V c.556G>T p.D186Y		c.86A>G p.N29S	c.*556 A>G	c.226+1G>A	
		1	2	3	4	5	6	7	8	9	10	11	12	13	Summary
Reference		(5)	(5)	(5)	(5)	(5)	(5)	(5)	(4)	(6)	(6)	(8)	(7)	This article	
Age		Child	9 month	1 day	Child	/	1 year	/	2 year	15 year	23 year	/	8 year	2.6 year	
Sex		M	M	M	F	F	F	M	/	M	F	/	M	M	
Growth															
Height	Short stature	+	+	+	+	+	+	+	+	+	+	−	−	+	11/13
Cardiovascular															
Heart	Dextrocardia	+	−	+	+	+	+	−	−	−	−	+	+	−	7/13
Vascular	Vertebral artery hypoplasia, unilateral	−	+	−	−	−	−	+	−	+	+	−	−	−	4/13
Respiratory															
Lung	Restrictive ventilatory defect	+	+	+	+	+	+	/	+	+	+	−	−	+	10/12
Chest															
Situs inversus		+	−	−	+	+	−	−	−	−	−	−	−	−	3/13
Short thorax		+	+	+	+	+	+	+	+	+	+	−	−	+	11/13
Rib deformity		+	−	+	+	+	+	+	+	+	+	−	−	+	10/13
Pectus excavatum		+	/	/	+	/	/	−	−	−	+	−	−	−	3/9
Abdomen															
Situs inversus		+	−	−	+	−	−	−	+	−	−	−	−	−	3/13
Spine															
Hemivertebrae		+	−	−	+	+	+	−	+	+	+	−	−	+	8/13
Butterfly vertebra		−	−	−	+	+	+	−	±	+	+	−	−	+	7/13
Abnormal odontoid process		−	−	−	−	+	+	−		+	+	−	−	−	4/13
Neurologic															
Spina bifida occulta		+	−	/	+	−	−	−	−	+	+	−	−	−	4/12
Myelomeningocele		+	−	/	+	−	−	+	+	−	−	−	−	−	4/12
Follow-up															
Alive		+	−	−	+	+	−	+	+	+	+	+	+	+	10/13

### 2.3. Splicing analysis of *HES7* c.226+1G>A in the minigene

**Figure 3:**
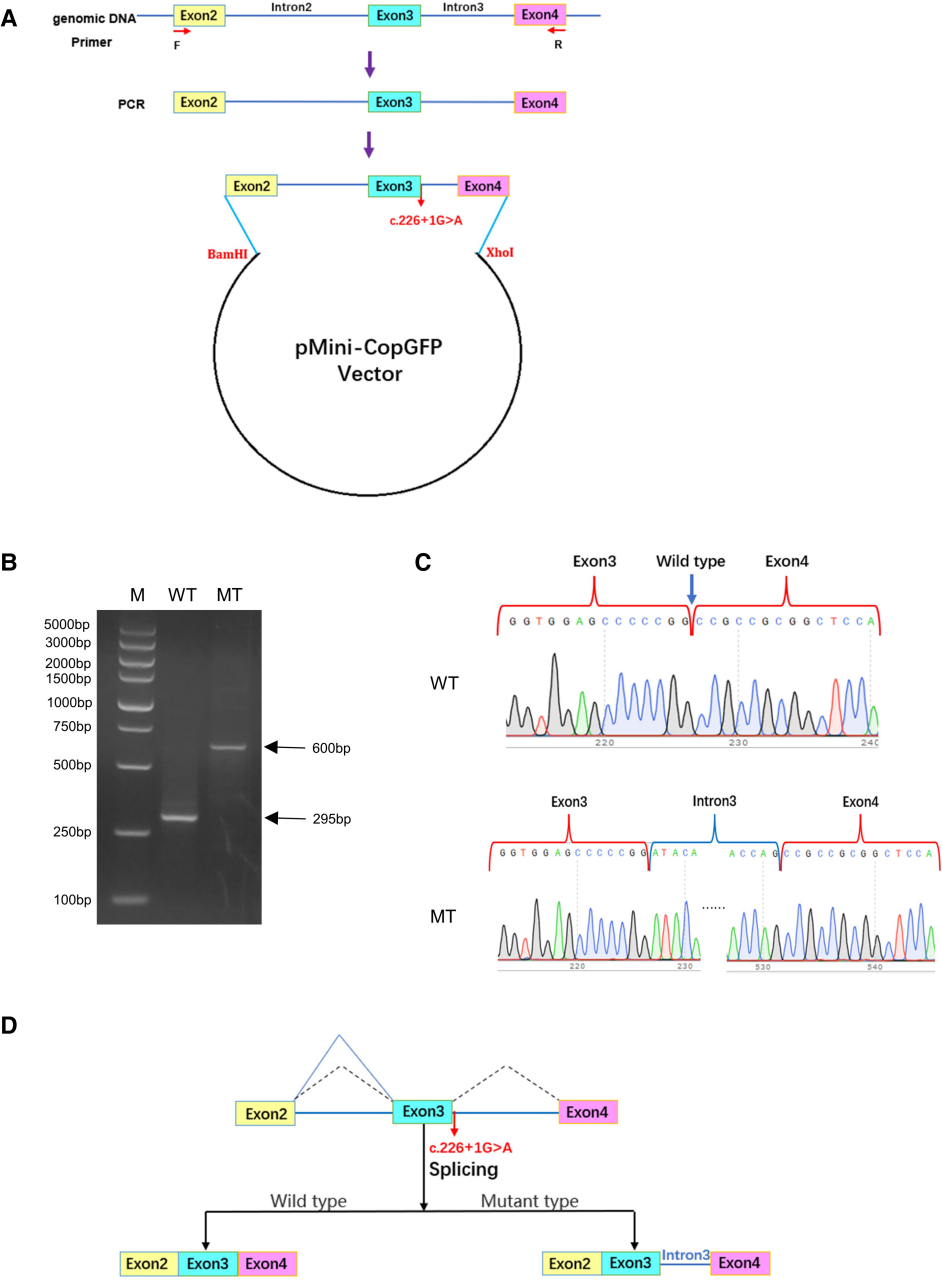
The effect of variant c.226+1G>A on the splicing of *HES7* mRNA*.* (**A**) Construction of minigene plasmids. (**B**) Agarose electrophoresis results of RT-PCR products. M, DNA marker DL5000; WT, 293 T cells transfected with wild-type plasmids; MT, 293 T cells transfected with mutant plasmids. (**C**) Sanger sequencing of gel-purified products. (**D**) Effect of variant c.226+1G>A on the splicing of *HES7* mRNA.

To investigate the effect of variant c.226+1G>A on the *HES7* gene, minigene plasmids were constructed and transfected into 293 T cells (Figure 3A). The wild-type DNA fragments were amplified using genomic DNA from healthy controls according to the manufacturer's instructions. Subsequently, mutant plasmids were constructed by PCR site-directed mutagenesis. Electrophoresis results revealed that the full-length amplification product was 295 bp in 293 T cells transfected with the wild-type minigene plasmids, while the amplification products were 600 bp in 293 T cells transfected with the mutant plasmids (Figure 3B). Sanger sequencing of gel-purified products demonstrated that the wild-type plasmid-transcribed mRNA sequences contained complete exon3 and exon4. However, the RT-PCR products of the mutant minigene plasmids also contained a complete intron3 sequence (Figure 3C), which proved that the variant *HES7* c.226+1G>A resulted in the retention of intron3 in the mutant mRNA (PVS1_strong, Figure 3D). The retention of intron3 would lead to a frameshift of *HES7* protein (*HES7* p.Ala76AspfsTer168, NP_001159439.1). However, we could not confirm this effect in the patient cells because his parents refused to do so. According to ACMG guidelines, we confirmed the variant to be likely pathogenic (PVS1_strong + PM2_ supporting + PP4).

## 3. Discussion

SCDO4 is a group of genetically heterogeneous diseases characterized by abnormal axial skeletal development. This condition is rarely encountered in clinical practice, with its main features being short stature (mainly short trunk), short chest, dyspnea, brain meningocele, spina bifida occulta, etc. In imaging examinations, hemivertebrae, butterfly vertebrae, rib fusion, spinal canal abnormalities, spinal segment defects or non-segmentation, and heart and large blood vessel malformations may be observed (13). SCDO4 is caused by homozygous or compound heterozygous variants in the *HES7* gene, which is located on chromosome 17 p13.1(Chr 17: 8,023,908–8,027,402) and contains four exons. The HES gene belongs to the basic helix-loop-helix (bHLH) superfamily, with domains including bHLH, orange domain, and the C-terminal conserved tetrapeptide WRPW. Seven HES genes can be found in humans (*HES1–7)*. Current studies suggest that *HES1*, *HES5*, and *HES7* are target genes of the Notch signaling pathway (13, 14), which are involved in the regulation of somite segmentation (15, 16).

Among them, *HES7* plays a major role in segmentation. *HES7* is expressed cyclically in the presomitic mesoderm (PSM) over a 2 h period (17). The absence of *HES7* leads to segmentation defects in mice (18). Moreover, severe disorders are observed in derivatives such as spine and rib tissue. *HES7* also regulates the cyclic expression of the Fgf signaling inhibitor Dusp4 and links Notch and Fgf oscillations in phase (19). Strikingly, the inactivation of Notch signaling abolishes the propagation but allows the initiation of *HES7* oscillation. In contrast, transient inactivation of Fgf signaling abolishes the initiation, whereas sustained inactivation abolishes both the initiation and propagation of *HES7* oscillation. Therefore, *HES7* oscillation is initiated by Fgf signaling and is propagated/maintained by Notch signaling (19). In addition, *HES7* also directly encodes a transcription repressor protein containing the bHLH domain, which is involved in the formation of the axial skeleton (20). The missense variants in the *HES7* gene usually impair the repress transcription ability. For example, both R25W and D186Y *HES7* do not repress transcription from promoters containing either N-boxes or E-boxes (4, 6). Hence, *HES7* is not only the direct target of the Notch signaling pathway but also a part of the negative feedback mechanism required to weaken the Notch signal (21). The *HES7* variant results in an interruption in the Notch signaling pathway and disrupted somite segmentation. Dysfunction of *HES7* proteins leads to the same malformations. Up to now, only six *HES7* variants have been reported to cause SCDO4, including four missense variants, one frameshift variant, and one variant in the 3′-UTR. In this article, a new type of *HES7* variant was identified. The novel variant *HES7* c.226+1G>A has been proven to influence the splicing of *HES7* mRNA.

This report is the first to present SCDO4 in a newborn in China. SCDO4 has a variety of clinical manifestations in the neonatal period, which can manifest as short stature (mainly short trunk), short chest, dyspnea, etc. A few severe cases of deformity can also cause death due to thoracic insufficiency and reduced lung capacity (22). Therefore, early treatment and intervention are needed. As the body develops, respiratory function gradually recovers. The proband developed normally after a 2.5-year follow-up, and the parents were satisfied with the development. Reportedly, patients who survive until adolescence and adulthood achieve normal functions (23). The proband's fetal examination revealed a disordered spinal arrangement and a low position of the medullary cone. He developed short stature, a short thorax, and difficulty breathing after birth. Chest x-ray and CT examinations revealed butterfly vertebrae, hemivertebrae, rib fusion, and vertebrae and tube enlargement, which are consistent with the description of SCDO4 in the OMIM database. The proband was further diagnosed as SCDO4 by genetic testing. Other children with SCDO4 may also present with cerebrospinal meningocele, neural tube defects, and heart and macrovascular malformations (5), which were not found in the proband of this article. The difference in clinical manifestations may be related to the heterogeneity of the disease phenotype (6, 24). Literature states that the expression of *HES1* and *HES5* genes is related to nephron development (25). Since the child had congenital hydronephrosis, the *HES7* variant may also be related to nephron development. However, this hypothesis requires further study.

In this case report, the effect of the novel *HES7* variant was verified by minigene assays, and a long-term follow-up and detailed auxiliary examinations were described. Nevertheless, this case report is also limited by the small number of SCDO4 cases.

In conclusion, a novel likely pathogenic *HES7* variant has been identified in a Chinese neonate with SCDO4. Our findings expand the genotype-phenotype knowledge of SCDO4 and provide new evidence for genetic counseling.

## Data Availability

Data of this study can be accessed after an approval application by the China National Gene Bank Database (https://db.cngb.org/). The project accession code is CNP0003966.
