# Supplementary material for: A novel homozygous HES7 splicing variant causing spondylocostal dysostosis 4: a case report
Source: Front Pediatr. 2023 Aug 25;11:1201999. doi: 10.3389/fped.2023.1201999 (PMC10485611; doi:10.3389/fped.2023.1201999)
Supplement: Supplementary file 1 [file Datasheet1.docx]

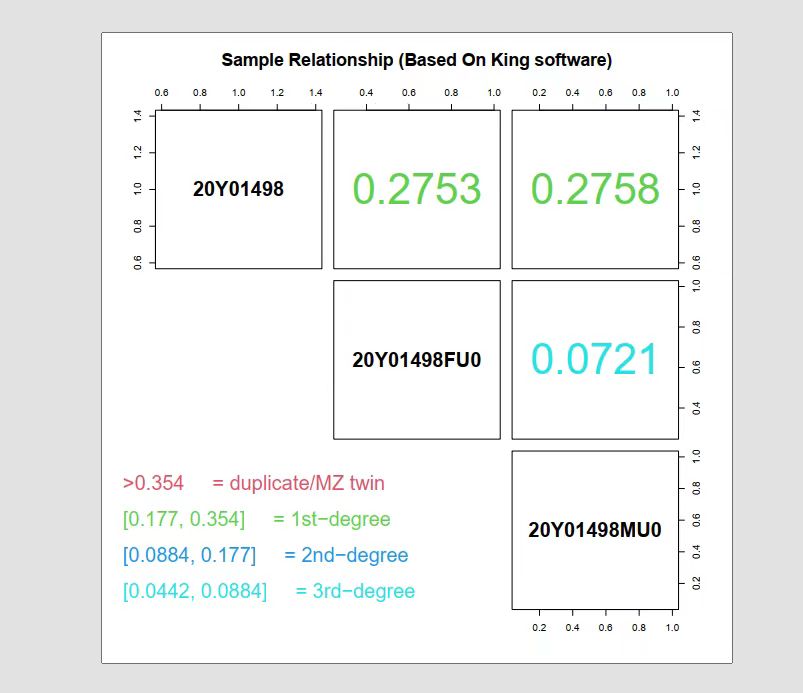


Figure S1. Kinship analysis with WES data. 20Y01498 stands for the proband; 20Y01498 FU0 stands for the father of the proband; 20Y01498 MU0 stands for the mother of the proband. The kinship values between the proband and his parents were 0.2753 and 0.2758 respectively. The kinship value between the couple was 0.0721 which indicated that they were third degree relatives. The result given by KING software is the estimation of kinship coefficient (φ) between two individuals. This coefficient represents the probability that two randomly selected alleles from the genotypes of the two individuals on a marker are identical by descent (IBD) to each other. The highest possible value for this coefficient is 0.5, which is observed in identical twins, and for parent-child pairs, φ=0.25.
